# Visual congruency of performers’ movements enhances vocal music reward through Mu entrainment

**DOI:** 10.1093/scan/nsaf089

**Published:** 2025-09-03

**Authors:** Lei Zhang, Yi Du, Robert J Zatorre

**Affiliations:** State Key Laboratory of Cognitive Science and Mental Health, Institute of Psychology, Chinese Academy of Sciences, Beijing 100101, China; Cognitive Neuroscience Unit, Montreal Neurological Institute, McGill University, Montreal, QC H3A 2B4, Canada; State Key Laboratory of Cognitive Science and Mental Health, Institute of Psychology, Chinese Academy of Sciences, Beijing 100101, China; Department of Psychology, University of Chinese Academy of Sciences, Beijing 100049, China; Chinese Institute for Brain Research, Beijing 102206, China; Cognitive Neuroscience Unit, Montreal Neurological Institute, McGill University, Montreal, QC H3A 2B4, Canada; Centre for Research in Brain, Language, and Music (CRBLM), Faculty of Medicine, McGill University, Montreal, QC H3G 2A8, Canada; International Laboratory for Brain, Music and Sound Research (BRAMS), Montreal, QC H2V 2J2, Canada

**Keywords:** music reward, audiovisual, sensorimotor integration, Mu wave, neural entrainment

## Abstract

There is emerging evidence that a performer’s body movements may enhance music-induced pleasure. However, the neural mechanism underlying such modulation remains largely unexplored. This study utilized behavioural, psychophysiological, and electroencephalographic data collected from 32 listeners (analysed sample = 31), as they watched and listened to vocal (Mandarin lyrics) and violin performances of pop music videos. None were familiar with Mandarin, and none had significant training in string instruments. Stimuli featured either congruent or incongruent audiovisual parings within the same instrument. We found that congruent visual movements, as opposed to incongruent ones, significantly increased both subjective pleasure ratings and skin conductance responses. While Mu-band power suppression occurred in the presence of visual movements regardless of congruency; congruent movements enhanced the coherence between the music envelope and Mu-band oscillations (so-called Mu entrainment). Effect sizes for both measures were greater for vocal than violin music, though no interaction was observed. Mediation analysis demonstrated that Mu entrainment to vocal music significantly mediated the visual modulation of music-induced pleasure and that this effect occurs primarily for familiar vocal rather than unfamiliar violin movements. In conclusion, our study provides evidence that congruent visual movements enhance music pleasure by promoting Mu entrainment, potentially through sensorimotor integration mechanisms.

## Introduction

Music, an art composed of auditory sequences, often provides profound pleasure (Zatorre 2024). However, this enjoyment frequently extends beyond sound, arising from the combined sensory experience of hearing music and observing performers’ gestures, whether live or via video. While the auditory features of music that generate pleasure are well-documented, the contribution of visual elements, such as body movements and facial expressions, to music-induced pleasure remains poorly understood.

Behavioural research has explored how visual movements influence music enjoyment. A meta-analysis found a medium effect size for increased pleasure when visual components were present (Platz and Kopiez 2012). Moreover, several studies suggest that visual cues can influence judgments about music performance more strongly than auditory cues (Tsay 2013, Griffiths and Reay 2018). These findings underscore the importance of visual elements in shaping musical experiences but call for objective evidence to elucidate the underlying physiological and neural mechanisms.

Skin conductance response (SCR), an indicator of sympathetic nervous system activity, measures phasic changes in skin electrical conductivity and reflects physiological arousal (Braithwaite et al. 2013). SCR is known to increase with increasing pleasure during music listening (Salimpoor et al. 2009, Mas-Herrero et al. 2014). However, whether visual observation of performers’ gestures enhances music-induced physiological arousal remains untested.

Music enjoyment also involves rhythmic synchronization between auditory stimuli and physical movements. The shared affective motion experience (SAME) model proposes that music is perceived not only as an auditory signal but also as intentional, hierarchically organized sequences of expressive motor acts (Overy and Molnar-Szakacs 2009). This indicates a role for auditory-motor integration in musical pleasure, which may depend on the mirror neuron system (MNS), or other mechanisms such as entrainment, or predictive coding (Hickok et al. 2011, Park et al. 2016). While listening to music, listeners may covertly or overtly mimic a performer’s gestures, facilitating the co-representation and sharing of musical experiences (Gagnon and Peretz 2003, Gomez and Danuser 2007, Michalak et al. 2009).

Mu activity, a brain rhythm typically within the 8–13 Hz frequency band, is a robust marker of motor activity and sensorimotor integration (Fox et al. 2016, Hobson and Bishop 2017, Jenson et al. 2020). Mu power suppression, originating in premotor areas and inferior parietal lobule, occurs during both action execution and observation (Fox et al. 2016, Hobson and Bishop 2017, Jenson et al. 2020, Bonini et al. 2022), and it has been linked to music perception (Mercadié et al. 2014, Ross et al. 2022, Wang et al. 2023). Importantly, this suppression amplifies when music listening is coupled with visual cues (Tanaka 2021). Moreover, recent advances in studying the neural tracking of continuous speech and music envelopes, including the Mu frequency band (Harding et al. 2019, Bröhl and Kayser 2021, Zuk et al. 2021, Aller et al. 2022), allow examination of how Mu oscillations entrain to the music envelope. This approach provides a novel framework to investigate how visual actions modulate music-specific Mu entrainment and its relationship with music pleasure.

**Figure 1.**
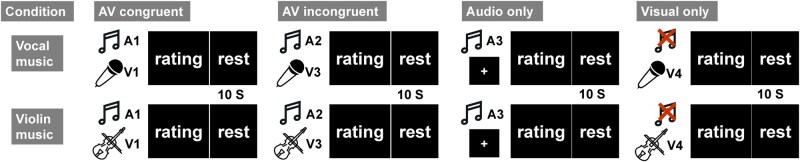
Experiment conditions. A denotes audio music stimuli and V denotes visual movements stimuli. AV congruent condition: Music stimuli and visual body movements stimuli were from the same video. AV incongruent condition: Music stimuli and visual body movements stimuli were from different videos. Audio-only condition: Only music stimuli was presented and a static cross was presented on the screen. Visual-only condition: Only silent visual body movement video was presented. Conditions were counterbalanced using a Latin square design. Music icons were sourced from https://www.svgrepo.com/svg/414475/sing, https://www.shareicon.net/music-violin-musical-instrument-orchestra-string-instrument-music-and-multimedia-815923, and https://uxwing.com/microphone-voice-icon/, all free and open-licensed sources.

In this combined behavioural, psychophysical, and electroencephalogram (EEG) study, we investigated how visual congruency influences music pleasure and its potential mediation by sensorimotor mechanisms. We exposed participants to vocal or violin music under four conditions: audio-visual congruent (AVc, matching music and visuals), audio-visual incongruent (AVic, audio tracks switched across videos), visual-only (VO), and audio-only (AO) (see Fig. 1). To assess music pleasure, we analysed subjective ratings and electrodermal activity (EDA) as an objective measure. Since Mu suppression reflects MNS activations regardless of cross-modal congruency (Crawcour et al. 2009, Tanaka 2021), we expected suppression in both audiovisual conditions. To assess neural processing of music signals, we used cerebral-acoustic coherence (CACoh) analysis (Peelle et al. 2013, Harding et al. 2019, Teng et al. 2024) to measure Mu entrainment, defined as coherence between Mu oscillations and the music envelope. We hypothesized that sensorimotor integration would be evident in significant visual modulation of Mu entrainment. Additionally, we analysed correlations between Mu activity (power suppression and entrainment), subjective ratings, and EDA and conducted mediation analyses to explore whether sensorimotor activity mediates the relationship between visual congruency and musical pleasure (VanderWeele 2016). Finally, we recruited participants naïve to violin playing to test whether sensorimotor integration depends on motor familiarity. According to the SAME model, visual congruency effects were predicted to be stronger for vocal music, given the universal familiarity with vocal tract movements compared to violin performance.

## Materials and Methods

### Participants

A priori power analysis in G*Power 3.1 (α  =  0.05, power = 0.8) indicated that 33 participants were needed to detect an effect size of *d* = 0.51 (Platz and Kopiez 2012) using a two-tailed paired *t*-test (Faul et al. 2009). Based on this and our design, we recruited 32 participants; one was later removed for fatigue. Additionally, EEG data from the first two subjects were discarded due to technical errors (their behavioural and EDA data were retained), and four subjects’ EDA data were excluded for non-responsiveness (Braithwaite et al. 2013). Therefore, the analysed sample size of behavioural, EEG and EDA data are 31, 29, and 27, respectively.

All participants (18 women; aged 18–33) were right-handed non-Mandarin speakers without neurological or psychiatric disorders. Except for one with two years of violin training, none had over 2 years’ experience with string instruments or vocal training, though some had training on other instruments. Detailed musical training histories for all participants are provided in Supplementary Table 1. All BMRQ scores exceeded 65 (Mas-Herrero et al. 2013), indicating no music anhedonia. Written consent was obtained according to the McGill University Institutional Review Board.

### Stimuli and procedure

Twenty naturalistic music pieces (10 vocal, 10 violin) were selected from YouTube and Bilibili. All pieces were Japanese or Chinese pop with Mandarin lyrics (Japanese pop lyrics were adapted into ­Chinese) to ensure unfamiliarity for non‐Mandarin-speaking ­Western listeners. Thus, any congruency effects arise solely from low-level cues (e.g. mouth, finger, and arm movements synchronized with sound). Videos featured only the performer against a static background; dynamic elements (e.g. rolling titles) were Gaussian‐blurred and clips trimmed to ∼80 seconds.

A pilot study with 13 participants rated music liking, and eight pieces were chosen based on these ratings and duration for the psychophysics/EEG experiment. Audio intensities were normalized (root mean square, 48 kHz), and videos rendered at 1280 × 720 pixels with a 30 Hz frame rate.

Clips were combined into four conditions (see Fig. 1):

- Audiovisual congruent (AVc): intact video with matching music;
- Audiovisual incongruent (AVic): audio tracks swapped across videos, pairing vocal with vocal and violin with violin (with pairs matched in duration, trimming videos as needed);
- Audio-only (AO): music with a static cross displayed centrally;
- Visual-only (VO): video presented without sound.

Each trial started with a 3-second static image (the first frame). The experiment comprised 8 blocks (violin/vocal × 4 conditions), with each block containing 4 trials. After each trial, participants rated (0–9) their liking, pleasure, arousal, and familiarity (if music was present) or visual performance (if the video was presented). After the rating responses, there was a 10 s inter-trial interval for potential movement-related artifacts to recover. Conditions were counterbalanced using a Latin square, and music type order balanced via an ABBA design (half the subjects received ABBA sequences, the other half BAAB).

### Behavioural data analysis

Behavioural responses for vocal and violin music were analysed separately using one-way repeated-measures analysis of variances (ANOVAs) to evaluate visual condition effects. Greenhouse–Geisser corrections were applied if sphericity was violated, and post hoc tests compared AVc vs. AO, AVic vs. AO, and AVc vs. AVic. When the Shapiro–Wilks test indicated non-normality, Wilcoxon signed-rank tests were used with Bonferroni correction for multiple comparisons. All analyses were conducted in R using bruceR (Bao 2021) and visualized with ggplot2 (Wickham 2009).

### EDA data acquisition and analysis

EDA data were recorded using a BIOPAC MP150 system and an EDA 100C amplifier. Electrodes with isotonic gel were attached to the volar surfaces of the distal phalanges of the right index and middle finger, with a sampling rate of 2000 Hz and a gain of 5 μS/V.

To keep all analysed trial lengths the same, only the first 77 seconds of each trial were analysed, as the shortest trial lasted 77 s. EDA signals were processed using Neurokit2 (Makowski et al. 2021) and custom Python scripts with default parameters to extract SCR counts and summed amplitudes; only SCRs peaking between 2 seconds after onset and the end of the music were considered (reflecting the ∼2 seconds latency). The AO condition served as baseline, and relative SCR differences for the AVc and AVic conditions were computed. Paired Wilcoxon signed rank tests compared SCR indexes between AVc and AVic for both vocal and violin music, given normality violations (Shapiro–Wilk *P* < .042).

### EEG data acquisition and processing

EEG data were acquired from 64 Ag/AgCl electrodes with the Brain Products actiCAP active electrode system, arranged according to the international 10–10 system, and included 2 electrooculogram (EOG) electrodes. Impedance of all electrodes was kept below 25 kΩ, sufficient for high-quality data collection according to the actiCAP system manual. All channels were referenced to the average of the left and right mastoid channels (M1, M2).

EEG data analysis was conducted using the Fieldtrip toolbox (Version 20230926, Oostenveld et al. 2011) and custom scripts for spectral and CACoh analysis. Details are introduced in the following sections. Similar to EDA data analysis, only the first 77 seconds of each trial were analysed. Raw EEG data were first bandpass filtered from 1 to 40 Hz and downsampled to 90 Hz. Independent component analysis (ICA) was performed to remove the artifacts from eye blinks, eye movements, and heartbeat. Mastoid channels and EOG channels were removed in the latter analysis.

### Spectral analysis

To investigate how visual performance affects Mu wave activity, spectral analysis was performed at each electrode. Preprocessed data were segmented into non-overlapping 1-second intervals. Power spectrum was computed and averaged across each condition using the function ‘ft_freqanalysis’ provided in FieldTrip. Frequencies of interest were from 1 to 40 Hz in steps of 0.5 Hz. Spectral power from 8 to 13 Hz was averaged to get the overall mu wave power under each condition.

### CACoh analysis

CACoh analysis quantified phase-locked neural responses to acoustic signals by computing the cross-spectrum coherence between neural signals and music amplitude envelopes (Peelle et al. 2013, Harding et al. 2019, Teng et al. 2024). Audio signals were processed through a bank of fourth-order gammatone filters with 16 logarithmically spaced centre frequencies from 100 to 4000 Hz, and the Hilbert transform extracted each band’s amplitude envelope. The 16 envelopes were then averaged, downsampled to 90 Hz, and bandpass filtered (1–40 Hz) to match EEG data. MATLAB’s ‘mscohere’ function estimated magnitude-squared coherence between EEG signals and the music envelope from 1 to 40 Hz (in 0.5 Hz steps), and coherence was averaged over 8–13 Hz to assess Mu wave entrainment.

### Cluster-based permutation test

The cluster-based permutation test in FieldTrip was performed to correct for multiple comparisons in spectral and CACoh analyses. Two-sided paired *t*-tests assessed differences in CACoh values between AVc and AVic conditions at each electrode. The sum of the *T*-statistics for statistically significant electrodes with a cluster was calculated as the cluster-level statistic. Five thousand Monte Carlo-based permutations were conducted to obtain the permutation distribution. The cluster-level alpha was set to 0.05.

### Regression and mediation analysis

Robust linear regression using the ‘robustbase’ package in R examined correlations between neural indices and music-induced pleasure (Maechler et al. 2025).

Within-participant mediation analysis with the ‘JSmediation’ package in R tested whether music processing mediated enhanced pleasure with valid visual movements (Yzerbyt et al. 2018). For vocal music, the mediator was the average CACoh difference (AVc vs. AVic), with visual congruency as the binary predictor and pleasure rating as the outcome. For violin music, the average CACoh difference served as the predictor, Mu suppression difference as the mediator, and SCR frequency or summed amplitude difference as the outcome. (Note that JSmediation reported original, not robust, regression coefficients.) Confidence intervals for the within-participant indirect effect were computed via Monte Carlo simulation with 5000 iterations, using an alpha threshold of 0.05.

## Results

### Congruent visual information increases subjective pleasure ratings to music

After each music piece under AVc, AVic, and AO conditions, participants gave their liking, pleasure, arousal, and familiarity ratings to the music pieces. After each video under the VO condition, participants gave their liking ratings to the video.

One-way repeated-measures ANOVA found that the main effects of visual conditions (AVc, AVic, and AO) on subjective pleasure ratings and liking ratings to music (*n* = 31) were significant only for vocal music (Pleasure: Vocal: *F*(2, 30) = 10.09, *P* < .001, $\eta_{p}^{2}$ = 0.402; Violin: *F*(2, 30) = 1.19, *P *= .166, $\eta_{p}^{2}$ = 0.113; Liking: Vocal: *F*(2, 30) = 6.97, *P *= .003, $\eta_{p}^{2}$ = 0.32; Violin: *F*(2, 30) = 2.23, *P *= .125, $\eta_{p}^{2}$ = 0.13). Similar negative findings for violin music were also found using Bayesian ANOVA analysis (Pleasure: Bayes factor = 1.12; Liking: Bayes factor = 0.48). No significant main effect of visual conditions was found on arousal and familiarity ratings (Arousal: Vocal: *F*(2, 30) = 0.35, *P *= .705, $\eta_{p}^{2}$ = 0.02; Violin: *F*(2, 30) = 1.93, *P *= .163, $\eta_{p}^{2}$ = 0.11; Familiarity: Vocal: *F*(2, 30) = 0.02, *P *= .985, $\eta_{p}^{2}$ = 0.00; Violin: *F*(2, 30) = 0.71, *P *= .501, $\eta_{p}^{2}$ = 0.05). We also perform two-way repeated ANOVA (visual conditions × music type) to examine whether the effect of visual conditions on liking and pleasure ratings are different between vocal and violin music. However, no significant interaction effect was found (Pleasure: *F*(2, 60) = 0.88, *P *= .403, $\eta_{p}^{2}$ = 0.03; Liking: *F*(2, 60) = 0.83, *P *= .829, $\eta_{p}^{2}$ = 0.01).

**Figure 2.**
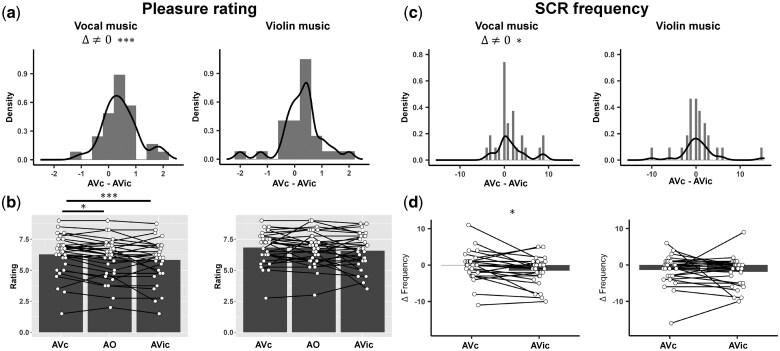
Bar, histogram, and density plots of subjective pleasure ratings and SCR frequency. (a, b) Subjective pleasure ratings were significantly higher in the AVc condition compared to the AO and AVic conditions for vocal music, but no differences were observed for violin music. (c, d) Relative SCR frequency was significantly higher in the AVc condition compared to the AVic condition for vocal music, with no significant differences for violin music. Note that, relative SCR frequency was calculated by subtracting the SCR frequency in the AO condition from the frequencies in the AVic and AVc conditions. *P < 0.05, ***P < 0.001.

As is shown in Fig. 2a and b, post hoc analysis found that congruent visual information (AVc condition) increased listeners’ pleasure ratings to vocal music compared to AVic condition (*t*(30) = 3.87, $P_{\text{corrected}}$ < .001, Cohen’s *d *= 0.79), but no significant effect was found for violin music (*t*(30) = 1.93, $P_{\text{corrected}\;}$ = .191, Cohen’s *d *= 0.28). Similar patterns were observed for liking ratings (vocal: *t*(30) = 3.53, $P_{\text{corrected}}$ = .004, Cohen’s *d* = 0.79; violin: *t*(30) = 2.09, $P_{\text{corrected}}$ = .137, Cohen’s *d *= 0.31).

To examine whether congruent visual information increased music-induced pleasure and liking ratings or incongruent visual information decreased music-induced pleasure and liking ratings, we compared pleasure and liking ratings between audiovisual conditions and AO condition (Wilcoxon signed rank tests were performed if the data violated the assumption of normality. Z-scores denote the statistics for Wilcoxon signed rank tests, and *r* values denote the effect size). Pleasure rating under AVc condition to vocal music was higher than that under AO condition (*Z *= 2.82, $P_{\text{corrected}}$ = .015, *r *= 0.51, Fig. 2b), while liking rating did not increase under AO condition (*t*(30) = 2.04, $P_{\text{corrected}}$ = .151, Cohen’s *d *= 0.42). However, pleasure rating and liking rating were not significantly different between AVic and AO conditions (pleasure rating: *t*(30) = 1.27, $P_{\text{corrected}}$ = .642, Cohen’s *d *= 0.64; liking rating: AO: *Z *= 2.00, $P_{\text{corrected}}$ = .138, *r *= 0.36). These results suggest that the impact of visual congruency on pleasure ratings to music can be attributed to the enhancement of these ratings by congruent visual information.

### Congruent visual information increases skin conductance responses during music listening

SCR frequency and amplitude were extracted from EDA data to investigate the effect of visual congruency on electrodermal activity. As shown in Fig. 2c and d, paired Wilcoxon signed rank test revealed that subjects showed significantly more SCRs under AVc condition than AVic condition when listening to vocal music (*Z *= 1.98, *P *= .048, *r *= 0.38), while no significant difference was found in violin music (*Z *= −0.67, *P *= .502, *r *= −0.13, Bayes factor = 0.24). Subjects also showed marginally larger summed SCR amplitude when listening to vocal music with congruent visual movement compared to AVic condition (*Z *= 1.87, *P *= .061, *r *= 0.36), while no effect was found in violin music (*Z *= −0.05, *P *= .960, *r *= −0.01, Bayes factor = 0.21). No significant effect of music type on SCR difference between AVc and AVic conditions was found (SCR frequency: *Z *= 1.28, *P *= .201, *r *= 0.25; summed SCR amplitude: *Z *= 1.40, *P *= .162, *r *= 0.27).

### Visual information suppresses Mu-band power independently of congruency

**Figure 3.**
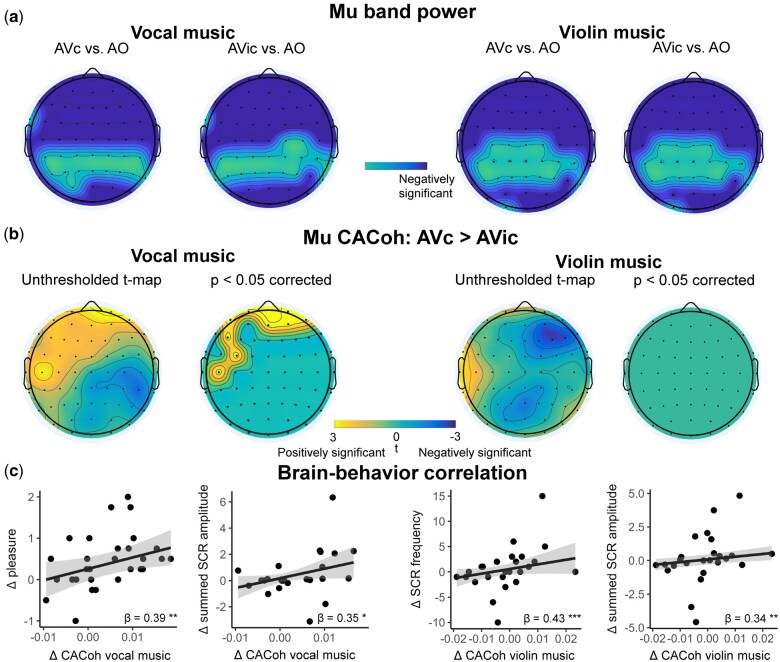
Results of Mu band power and Mu CACoh analyses. (a) Mu-band power was suppressed under AVc and AVic conditions compared to AO condition for both vocal and violin music. (b) Mu entrainment to music, as calculated by cerebral-acoustic coherence (CACoh) between Mu-band signal and music amplitude envelope, was enhanced under AVc condition compared to AVic condition only for vocal music. (c) Enhanced neural entrainment to music was correlated to greater subjective pleasure ratings and SCR indices for both vocal and violin music. *P < 0.05, **P < 0.01, ***P < 0.001.

Mu-band power was extracted for each visual condition to investigate whether the presence of visual information inhibited Mu-band power and whether visual congruency modulated Mu-band power during music perception. As is shown in Fig. 3a, Mu-band power under conditions with visual information was significantly inhibited compared to AO condition at almost all frontal, temporal, and occipital electrodes (AVc vs. AO, AVic vs. AO, $P_{\text{fwe}}$ < .05) for both vocal and violin music. However, we did not find any electrodes that showed a significant difference in Mu power between AVc and AVic conditions.

Additionally, Mu-band power was significantly suppressed at all central and frontal electrodes under AVc condition compared to AO condition during vocal music perception. And no correlation was found between mu suppression and behavioural measures in vocal music. However, for violin music, Mu-band power under AVc condition did not show significant suppression at Cz, C1, C2, and C3 electrodes, which typically showed Mu suppression when a person performed or observed an action (Hobson and Bishop 2017).

### Visual congruency modulates Mu entrainment to vocal music

In addition to Mu-band power, CACoh analysis was performed to investigate the effect of visual congruency on the neural entrainment to the music signal in the Mu band. As shown in Fig. 3b, Mu entrainment for vocal music was significantly greater at frontal electrodes (FP1, FP2, AF7, AFz, AF4, AF8, F5, FC3, and C5) under AVc condition than AVic condition ($P_{\mathrm{fwe}}$ < .05). However, no electrodes showed a significant effect of Mu entrainment for violin music.

Robust linear regression analysis was conducted to investigate the association of the music Mu entrainment difference at electrodes that showed a significant visual congruency effect with the SCR difference, and with subjective rating difference between AVc and AVic conditions. We found that the average increased Mu entrainment across significant channels could significantly predict the increased pleasure ratings in vocal music (Fig. 3c, β  =  0.39, *P *= .001). Also, increased Mu entrainment could predict larger summed SCR amplitude for vocal music (β  =  0.35, *P *= .034).

To rule out the possibility that the visual congruency effect was related to differences in attentional engagement, we further examined the alpha power in the occipital and parieto-occipital electrodes, which is closely associated with the strength of attention (Hobson and Bishop 2017, Deng et al. 2020, Klatt et al. 2022). We found no significant differences between the two visual conditions, even using a loose statistical threshold (all *P* > .2). Therefore, there is no evidence to suggest a difference in attention between these two audiovisual conditions.

### Greater Mu entrainment to violin music with congruent visual information is correlated to increased music-induced pleasure and Mu suppression

We did not find a visual congruency modulation effect to violin music-induced pleasure and neural entrainment generally. However, considering individual differences in audiovisual violin music perception, we hypothesized that subjects who showed greater Mu suppression or Mu entrainment to music under visual congruent condition compared to visual incongruent condition could demonstrate greater music-induced pleasure. Therefore, we performed robust linear regression analysis to examine whether Mu power difference across electrodes that showed significant suppression in audiovisual vocal music but not in audiovisual violin music (Cz, C1, C2, and C3) or average Mu entrainment difference at electrodes that showed greater neural entrainment to vocal music under AVc condition (FP1, FP2, AF7, AFz, AF4, AF8, F5, FC3, and C5) could predict violin music-induced pleasure difference. And we also examined the relationship between the average Mu entrainment difference and Mu power difference.

Robust linear regression results showed that greater Mu entrainment under AVc condition compared to AVic condition predicted greater summed SCR amplitude, SCR frequency, and Mu suppression for violin music (Fig. 3c, summed SCR amplitude: β  =  0.34, *P *= .005; SCR frequency: β  =  0.43, *P *< .001; Mu suppression: β = −0.36, *P *< .001).

Additionally, greater Mu suppression under AVc condition compared to AVic condition was correlated with increased subjective arousing ratings (β = −0.16, *P *= .021), and longer musical training years could marginally predict greater Mu suppression (β = −0.15, *P *= .065).

### Increased Mu entrainment to vocal music mediates increased music-induced pleasure rating with congruent visual information

**Figure 4.**
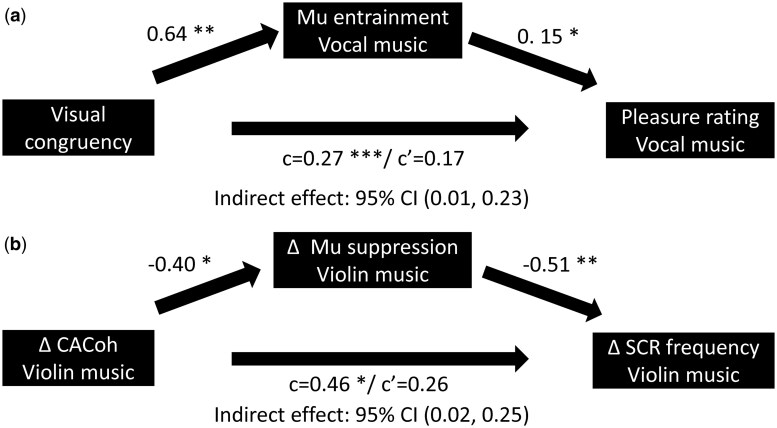
Within-subject mediation analysis results. (a) Mu entrainment to vocal music mediated the visual modulation on subjective pleasure ratings. (b) Enhanced Mu power suppression to violin music under AVc condition was the significant mediator of the correlation between enhanced Mu entrainment and greater SCR frequency to violin music under AVc condition compared to AVic condition. *P < 0.05, **P < 0.01.

Since we found that visual congruency modulated both Mu entrainment to music and music-induced pleasure to vocal music, we explored whether enhanced entrainment to music mediates increased music-induced pleasure under AVc condition. The electrodes that showed significantly greater Mu entrainment to vocal music were included in the within-subject mediation analysis. As shown in Fig. 4a, we found a significant indirect effect of visual congruency on subjective pleasure ratings through increased mean music Mu entrainment value across all significant electrodes (visual congruency → average Mu entrainment value → subjective pleasure rating, 95% CI, 0.01 to 0.23). Additionally, we found a full mediation effect of the mean Mu entrainment, since visual congruency was not correlated with pleasure rating after controlling the mean Mu entrainment (from β  =  0.27, *P *< .001 to β  =  0.17, *P *> .05). These results indicate that Mu entrainment fully explained the visual modulation on subjective pleasure rating.

### Increased Mu suppression mediates the association between increased Mu entrainment to violin music and violin music-induced SCR change

In violin music, we discovered a significant correlation between the difference in Mu entrainment, SCR frequency, and the summed SCR amplitude difference under AVc and AVic conditions, as well as the difference in Mu suppression. We thus conducted the mediation analysis to explore further relationships between neural and psychophysiological indices. As shown in Fig. 4b, we found a significant full mediation effect of Mu suppression between Mu entrainment to violin music and SCR frequency difference (Δ Mu entrainment → Δ Mu suppression → Δ SCR frequency, 95% CI, 0.02 to 0.25, from β  =  0.46, *P *= .019 to β  =  0.17, *P *> .05). These results suggest that increased Mu suppression fully explained the relationship between Mu entrainment to violin music and the physiological difference. However, the mediation effect was not significant when Mu entrainment was set as the mediator (Δ Mu suppression→ Δ Mu entrainment → Δ SCR frequency, 95% CI, −0.26 to 0.05). Also, we did not find a significant mediation effect when summed SCR amplitude difference was set as the dependent variable (Δ Mu entrainment → Δ Mu suppression → Δ summed SCR amplitude, 95% CI, −0.03 to 0.40; Δ Mu suppression→ Δ Mu entrainment → Δ summed SCR amplitude, 95% CI, −0.30 to 0.10).

## Discussion

This study shows that congruent performer movements increase listeners’ pleasure in vocal music, both behaviourally and physiologically, using naturalistic music pieces. The enhancement appears to arise from facilitated sensorimotor integration, as indicated by increased Mu entrainment to the music envelope in the presence of congruent visual vocal movements. Importantly, Mu entrainment fully mediated the effect of visual congruency on music pleasure ratings for vocal music, while no such effects were observed for violin music among non-violin-playing participants. This suggests that sensorimotor integration may be more effective for movements familiar to the listener, although further study is needed given the lack of a significant interaction. These results highlight that meaningful visual information enhances music reward potentially through sensorimotor integration.

Several behavioural studies have documented increased music performance appreciation when listeners view congruent performer movements (Vines et al. 2006, Vines et al. 2011, Platz and Kopiez 2012, Tsay 2013, Griffiths and Reay 2018, Eaves et al. 2020). Consistent with this phenomenon, we found that listeners’ subjective pleasure ratings for vocal music were enhanced under congruent visual conditions. Furthermore, SCRs, which are well-established objective indicators of arousal and reward in response to music (Grewe et al. 2007, Salimpoor et al. 2009, Zatorre and Salimpoor 2013, Mas-Herrero et al. 2014, Martínez-Molina et al. 2016, Solberg and Dibben 2019) increased in both frequency and amplitude when the visual information was congruent, supporting the hypothesis that congruency between the performer’s movements and the music significantly heightens musical pleasure.

The role of the motor system in music-induced pleasure remains underexplored. A recent meta-analysis failed to find a significant association between cortical motor areas and music-induced pleasure (Mas-Herrero et al. 2021). Previous fMRI studies have shown that the motor system is involved in rhythmic information processing (Gordon et al. 2018), and there are significant correlations between cortical and subcortical motor activities and beat-related pleasure (Kornysheva et al. 2010, Trost et al. 2014, Matthews et al. 2020). In vocal music perception, visual cues from performers offer important information about timing, pitch, and amplitude, potentially enhancing predictability and reducing uncertainty. Previous studies investigating how music surprisal and entropy interact with pleasure rating found that music with intermediate levels of complexity (surprisal) and uncertainty (entropy) received higher pleasure ratings (Cheung et al. 2019, Gold et al. 2019). Since participants in the current study were not familiar with the music used in this experiment (0–9 rating, song: mean familiarity = 1.03; violin: mean familiarity = 2.27), we speculate that congruent visual information may lower uncertainty by providing clearer, more predictive cues, thereby leading to greater musical pleasure.

Mu-band power, a marker of sensorimotor integration, is typically suppressed during the execution of actions or observation of others’ actions (Hobson and Bishop 2017). Beyond visual movements, the motor system is also involved in speech processing (Murakami et al. 2015, Park et al. 2016, Zhang and Du 2022, Zhang et al. 2023), highlighting its multimodal sensitivity. Consistent with previous studies (Crawcour et al. 2009, Tanaka 2021), our EEG results found Mu power suppression during visual movement observation, regardless of cross-modal congruency. However, Mu suppression during naturalistic music perception primarily reflects the general state of the motor system and does not necessarily capture the brain’s specific processing of musical signals. To overcome this limitation, we directly measured the coherence between Mu-band oscillations and the music envelope signal, positing that if sensorimotor integration contributes to music perception, then congruent visual movements would enhance Mu entrainment. Supporting this hypothesis, we found greater coherence between Mu-band activity and the music envelope in the presence of congruent visual movements, but only during vocal music, not violin music. This effect was localized to sensorimotor and frontal electrodes, distinguishing it from attentional effects typically associated with alpha-band activity in occipital and parietal electrodes (Fox et al. 2016, Hobson and Bishop 2017, Deng et al. 2020, Klatt et al. 2022), thereby substantiating the sensorimotor origin of Mu activities in the current study. Moreover, mediation analysis demonstrated that Mu entrainment fully mediated the relationship between visual congruency and pleasure ratings for vocal music, highlighting the role of sensorimotor integration in enhancing musical rewards through meaningful visual cues.

Note that none of the participants in this study could play the violin, while all could sing to some extent. Recent neuroimaging and neural modulation studies have demonstrated the motor system’s importance for speech perception, especially under challenging conditions (Du et al. 2014, Zhang and Du 2022, Liang et al. 2023, Zhang et al. 2023, Zhang et al. 2025). While motor system involvement is not essential for music listening, prior studies show that it becomes more prominent when people have acquired knowledge of playing the musical instrument (Lahav et al. 2007, Herholz et al. 2016, Gordon et al. 2018, Srinivasan et al. 2020). Musical training experiments have further found that sensorimotor integration increases during music listening after training (Zatorre et al. 2007, Herholz and Zatorre 2012, Herholz et al. 2016, Wollman et al. 2018, Srinivasan et al. 2020). These findings may explain why listeners benefited from observing vocal music production. With knowledge of vocal movements, they could extract more information from congruent visual cues. Conversely, participants’ lack of familiarity with violin playing likely reduced their ability to derive meaningful information from violin-specific motions, rendering visual congruency less influential on violin music perception.

Although we did not find a significant modulation of visual congruency on violin music processing or pleasure, some participants may still have gathered information from violin-playing movements, such as bow movements correlated with note onset and dynamics. Regression analysis showed that increased Mu entrainment was significantly associated with higher pleasure ratings for violin music, as well as for vocal music, suggesting that listeners exhibiting stronger music-specific Mu responses experienced greater pleasure when visual movements were congruent. These findings highlight the need for future studies to more precisely examine how familiarity and motor expertise shape audiovisual integration in music perception.

This study has several limitations. The naturalistic stimuli did not allow for isolating general body movements (e.g. swaying) from the fine motor actions involved in violin playing; future research could use motion capture combined with techniques such as Partial Information Decomposition to disentangle the kinematic and auditory contributions of different motion components. Additionally, while we manipulated audiovisual congruency, we did not address synchronization between audio and visual streams, so subsequent work should investigate how timing impacts music-induced pleasure. We specifically excluded individuals with violin or vocal training to test whether sensitivity to movements associated with those forms of musical expression would extend to populations without such expertise. However, this design choice limits our ability to interpret whether familiarity with instrument-specific actions enhances these effects and the generalizability of the findings. Future studies should include participants with such expertise to explore how musical training influences the visual impact on music perception. Moreover, our design precludes establishing a causal role for Mu entrainment in musical pleasure, which could be explored using neural modulation techniques like rhythmic TMS or tACS. Finally, the limited spatial resolution of EEG hinders precise localization of the brain areas involved in music entrainment and pleasure, suggesting that high-resolution neuroimaging methods like MEG or fMRI would be beneficial for revealing functional connectivity between auditory and motor regions.

In sum, this study provides empirical evidence that vocal music-induced pleasure is modulated by observing performers’ body movements, as reflected in both subjective ratings and skin conductance responses. Performers’ movements convey information about music production, likely enhancing sensorimotor integration during music perception. This integration may improve predictions and reduce uncertainty—factors associated with greater pleasure (Zatorre 2024). These findings also have practical implications for music education and the music production industry: aligning visual elements with musical actions can enhance listener engagement and emotional responses.

## Supplementary Material

nsaf089_Supplementary_Data

## Data Availability

All data needed to evaluate the conclusions in the paper are included, and the behavioural, EDA, and EEG data supporting these findings are available on OSF: https://osf.io/stw2u/.
