# Supplementary material for: Visual congruency of performers’ movements enhances vocal music reward through Mu entrainment
Source: Soc Cogn Affect Neurosci. 2025 Sep 3;20(1):nsaf089. doi: 10.1093/scan/nsaf089 (PMC12640236; doi:10.1093/scan/nsaf089)
Supplement: nsaf089_Supplementary_Data [file nsaf089_supplementary_data.docx]

Supporting Information

Supplementary Table 1. Musical training information for all participants.

|  | Year of training | Instruments |
| --- | --- | --- |
| subj1 | 2 | piano |
| subj2 | 1.5 | piano |
| subj3 | 0 |  |
| subj4 | 0 |  |
| subj5 | 2 | violin |
| subj6 | 0.25 | piano |
| subj7 | 13 | piano, saxophone, clarinet |
| subj8 | 3 | piano |
| subj9 | 0 |  |
| subj10 | 12 | piano, flute |
| subj11 | 0 |  |
| subj12 | 0 |  |
| subj13 | 7 | guitar |
| subj14 | 4 | piano |
| subj15 | 15 | piano |
| subj16 | 0 |  |
| subj17 | 0 |  |
| subj18 | 0 |  |
| subj19 | 0 |  |
| subj20 | 16 | guitar, piano, voice |
| subj21 | 0 |  |
| subj22 | 3 | trumpet |
| subj23 | 6 | piano |
| subj24 | 0 |  |
| subj25 | 0 |  |
| subj26 | 0 |  |
| subj27 | 0 |  |
| subj28 | 0 |  |
| subj29 | 1 | harmonium |
| subj30 | 0 |  |
| subj31 | 17 | piano, guitar |
| subj32 | 14 | clarinet |
